# Fracture of the Cranium

**Published:** 1871-08

**Authors:** Theo. W. Stull

**Affiliations:** Marengo, Ill.


ARTICLE V.—*Fracture of the Cranium.* By THEO. W. STULL, M.D., Marengo, Ill.

Seeing a case of fracture of the cranium reported not long since, reminded me of the following—to me—interesting case; and thinking, perhaps, that it might be interesting to others as well, is my reason for reporting it.

On the 11th of April, 1870, Dr. J. W. Green requested me to see a patient with him at his office. Said patient was a stout, healthy lad—Dennis S.—about 15 years of age, of whom the father gave the following history:

Ten days before, while leading a horse to water, he had been struck on the top of the head by a dull heel cork of the animal's fore foot, it rearing and striking in play. The lad fell partially down, but immediately recovered himself, pursued and caught the horse. During the following week he plowed considerably, and worked about as usual, without manifesting any particular symptoms of illness. One week from date of injury, he was attacked with spasmodic contractions of the muscles of the limbs of the right side, while at work. By a brisk friction of the muscles the spasmodic action was relieved, but a partial paralysis remained. The father now took the boy to Harvard, to his physician—who was then sick, and since dead—and, after describing the case to him—the boy remaining out in the wagon—he was advised to take his son to Woodstock or Marengo, the doctor remarking that there was no one in Harvard who could do him any good. Not satisfied with this, the father took the boy to another physician, who claims to be a regular, and he examined the case, told the father to keep a plaster over the slight wound of the scalp, gave him a vial of medicine, and told him the lad would be well in a week. Two days later, the father, not satisfied with the boy's progress toward a convalescence, decided to bring the lad to Marengo, to Dr. Green, which he did on the 11th, a distance of fifteen miles, in a lumber wagon, and on a hot spring day. After a short examination, we informed the father that the skull was fractured, and that an operation was necessary. After informing him of the gravity of the case, we appointed the next day to go to the father's house and operate, which we did. The lad could still walk with a dragging movement of the right limb. The head was now closely shaven for some distance around the scalp wound, chloroform was administered to anæsthesiæ, and Dr. Green commenced the operation by making a V shaped incision, apex to the front through the skin and tissues, down to the cranium. The shape and size of the fracture of the outer table was of the shape and size of a well-worn heel cork, and was situated about 1¼ inches posterior to the coronal, and about half an inch to the left of the sagittal suture. The fracture of the inner table was in several pieces, and imbedded into the brain substance. The Doctor succeeded with but little difficulty in removing all the fragments, the aggregate of which formed a piece three-fourths of an inch wide and one inch in length. Brain matter escaped to the amount of the size of a large hazel nut. The hæmorrhage was quite profuse. After thoroughly cleansing the wound and staunching the blood, the wound was closed with adhesive straps, a cold water compress applied, and a continued use of the cold water dressing directed. The patient rallied well from the effects of the chloroform, and was placed in bed. A light diet was directed. The third day Dr. Green again visited this patient, and found him sitting up and able to walk about as he chose, the paralysis being gone. The Doctor now dismissed the case.

The last fall the father again visited our place. He informed

Dr. Green that the tenth day after the operation the boy went to plowing, and continued well through the season.

It is at least passing strange that the boy should receive so severe an injury of the skull without experiencing scarcely a noticeable shock from the concussion, and also that with this amount of injury to the brain matter, there should result so little inconvenience to the nervous system.

